# Use of gene-editing technology to introduce targeted modifications in pigs

**DOI:** 10.1186/s40104-017-0228-7

**Published:** 2018-01-29

**Authors:** Junghyun Ryu, Randall S. Prather, Kiho Lee

**Affiliations:** 1 Department of Animal and Poultry Sciences, Virginia Tech, 175 W. Campus Drive, Blacksburg, VA 24061 USA; 2 Division of Animal Science, University of Missouri-Columbia, 920 East Campus Drive, Columbia, MO 65211 USA; 3 National Swine Resource and Research Center, University of Missouri-Columbia, Columbia, MO 65211 USA

**Keywords:** CRISPR/Cas9, Gene-editing, Genetic engineering, Knock-in, Knockout, Pig, TALEN, ZFN

## Abstract

Pigs are an important resource in agriculture and serve as a model for human diseases. Due to their physiological and anatomical similarities with humans, pigs can recapitulate symptoms of human diseases, making them a useful model in biomedicine. However, in the past pig models have not been widely used partially because of the difficulty in genetic modification. The lack of true embryonic stem cells in pigs forced researchers to utilize genetic modification in somatic cells and somatic cell nuclear transfer (SCNT) to generate genetically engineered (GE) pigs carrying site-specific modifications. Although possible, this approach is extremely inefficient and GE pigs born through this method often presented developmental defects associated with the cloning process. Advancement in the gene-editing systems such as Zinc-Finger Nucleases (ZFNs), Transcription activator-like effector nucleases (TALENs), and the Clustered regularly interspaced short palindromic repeat (CRISPR)/CRISPR-associated 9 (Cas9) system have dramatically increased the efficiency of producing GE pigs. These gene-editing systems, specifically engineered endonucleases, are based on inducing double-stranded breaks (DSBs) at a specific location, and then site-specific modifications can be introduced through one of the two DNA repair pathways: non-homologous end joining (NHEJ) or homology direct repair (HDR). Random insertions or deletions (indels) can be introduced through NHEJ and specific nucleotide sequences can be introduced through HDR, if donor DNA is provided. Use of these engineered endonucleases provides a higher success in genetic modifications, multiallelic modification of the genome, and an opportunity to introduce site-specific modifications during embryogenesis, thus bypassing the need of SCNT in GE pig production. This review will provide a historical prospective of GE pig production and examples of how the gene-editing system, led by engineered endonucleases, have improved GE pig production. We will also present some of our current progress related to the optimal use of CRISPR/Cas9 system during embryogenesis.

## Background

Genetically engineered (GE) animals have been an essential resource in advancing the biomedicine field. Traditionally, GE mouse models have been widely used because of the ability to engineer their genome through gene targeting and produce GE mice carrying site-specific modifications by using embryonic stem (ES) cells [1]. The mouse models are advantageous as they can be effectively managed and bred due to their size and life span. However, these mouse models cannot represent symptoms of certain human diseases, probably because of anatomic and physiological differences between mice and humans. The pig models, on the other hand, can closely recapitulate the phenotype of many human diseases due to similar physiology, anatomy, immunology, and metabolic features compared to humans [2, 3]. For instance, GE pigs carrying mutated *CFTR* genes present similar symptoms of human CFTR patients [4], where GE *CFTR* mouse models do not show these phenotypes. Although the benefit of using large animal models, such as pigs, in biomedicine is well-recognized, one of the major problems of applying pig models in biomedicine is inefficiency in genetic engineering technology. Because of the lack of ES cells, traditional GE pigs are produced by introducing targeted modifications in somatic cells, then generating GE pigs through somatic cell nuclear transfer (SCNT). A few breeding steps are required to generate animals with homozygous mutations because only heterozygous mutated pigs were produced due to the low efficiency of traditional gene targeting. However, considering the gestation period of pigs and time to reach sexual maturity, generating homozygous GE pigs has been expensive and can take several years. Development of engineered endonucleases now allows us to overcome these shortcomings. The endonucleases have shown to increase the targeting efficiency significantly and multiallelic modifications can be introduced into somatic cells [5, 6]. In addition, direct injection of engineered endonucleases, for example CRISPR/Cas9, can disrupt multiple genes during embryogenesis [7–9]. The use of GE pigs has been concentrated in biomedicine due to available resources; however, the development of engineered endonucleases now expands their application beyond biomedicine. This review will focus on the historical aspects of pig models and how recent technologies have changed the potential uses of pig models in research.

## Historical approach of generating genetically engineered pigs

For a complete understanding of how a biological system works it is necessary to dissect and manipulate the system. That manipulation can include altering the genome. It should be noted that the genomes of domestic animals, pigs in particular, have been altered by man for millennia. Selective breeding of cattle, for example, has resulted in animals that are more suited to milk production or to meat production. In pigs over just the past 50 years we have gone from a ‘lardy’ type pig to a highly productive, very prolific, long lean animal. These changes in phenotype in both cattle and pigs have been brought about by selection of natural variation already present in the population. Genetic engineering is the logical next step. The beginning of intentional genetic change mammals was reported as long ago as 1971 [10]. While viral mediated transgenesis was developed first [11], a more widely used genetic engineering technology was that of pronuclear injection [12]. Pronuclear injection was technically easier than viral transduction and very large constructs could be integrated into the genome. Pronuclear injection is a powerful tool to ask questions about the function of transgenes. Theoretically, any protein can be expressed at any level, compatible with development, in any cell type. Pronuclear injection does, however, have limitations that include: lack of control over the site of integration (e.g. possibly introducing an insertional knockout such as situs inversus [13], and lack of control of the number of copies of the gene that integrate. Nevertheless, pronuclear injection was used to create numerous lines of pigs [14–16]. A more precise method of altering the genome was introduced with homologous recombination [17]. In mice, the homologous recombination technology was used in conjunction with the newly discovered embryonic stem cells that could contribute to the germ line. This technology continues to be used in an attempt to knockout every single gene in the mouse [18]. Knock out of a gene unambiguously defines its function, and thus a better understanding of how the biological system functions can be obtained.

Unfortunately, despite concerted efforts [19–22], a suitable stem cell line has not been identified in the pig. Thus making a knockout in pigs was problematic. In parallel to the development of the embryonic stem cell technology, nuclear transfer was developed in domestic animals (sheep [23], cattle [24], pigs [25]. Extension of these early experiments that used donor nuclei from cleavage stage embryos led to later stages of embryos such as the inner cell mass of blastocyst stage embryos [26] to fetal derived fibroblast cells [27] to adult derived cells [28]. Since somatic cells could be grown in vitro and then used for somatic cell nuclear transfer, genetically engineering them prior to nuclear transfer would result in that particular genetic modification in the offspring. This was first demonstrated in sheep [29, 30] and then in pigs by introduction of a transgene [31] and the knockout of an endogenous gene [32]. To date a large number of transgenes have been added to pigs and a large number of genes have been knocked out [3, 33, 34]. However, efficiency of the entire procedures was extremely poor until the development of engineered endonucleases such as Zinc-Finger Nucleases (ZFNs), Transcription activator-like effector nucleases (TALENs), and Clustered regularly interspaced short palindromic repeat (CRISPR)/CRISPR-associated 9 (Cas9) system.

## Mechanism of engineered endonucleases

To-date, three types of engineered endonucleases, ZFNs, TALENs, and CRISPR/Cas9 system, have been developed to facilitate genetic engineering process. The specific content of each engineered endonuclease will be introduced in following paragraphs. All three engineered endonucleases have DNA binding ability and utilize DNA double-strand break (DSB) as a means to introduce targeted modifications into the genome. The endonucleases are designed to introduce DSBs on a specific location in the genome as a molecular DNA scissors. Then, the DSBs will trigger the endogenous DNA repair processes, which can then introduce targeted modifications. The DSB, created by these engineered endonucleases, needs to be repaired and would otherwise be lethal to cells. During the DSB repair, the presence of template DNA can induce site-specific recombination through homology-directed repair (HDR). If no donor DNA is available, the DSB is repaired by non-homologous end joining (NHEJ), which often introduces short DNA insertions or deletions, so called indels, that create targeted gene knockouts because the indels can induce a frameshift of amino acid codons, which often results in the formation of a premature stop codon [35]. In general, the frequency of NHEJ is known to be higher compared to that of HDR in most cell types [36].

## Use of gene-editing technology in GE pig production

### Gene targeting in somatic cells for GE pig production

#### Zinc finger nucleases (ZFNs)

Zinc-finger nucleases were the first engineered endonucleases developed by combining DNA recognition ability of zinc-finger (ZF) protein and endonuclease property of *FokI* enzyme. A ZF protein motif, first identified from *Xenopus* oocytes while studying the structure of factor IIIA [37], can recognize and bind to three nucleotides, and these ZF proteins can be connected to recognize a longer DNA sequence. Then, these ZF proteins were fused with the chimeric restriction enzyme, *Fok*I, to generate ZFNs [38], which was the beginning of genetic engineering by engineered endonucleases. The incorporation of endonucleases was an essential component of a gene-editing system because previous studies of DNA repair using *I-Sce*I clearly demonstrated that DSBs could enhance the frequency of HR [39–45]. The first demonstration of ZFNs as an effective gene-editing system in mammal was in human cells [5]. The frequency of gene targeting in this study was over 18% without any selection step; compared to a conventional gene targeting approach, there was 1000-fold increase in targeting efficiency. The study also showed that ZFNs could be successfully used to introduce site-specific mutations through HDR by activating endogenous homologous recombination (HR) pathway.

In 2011, three types of GE pigs were generated using the ZFN technology. The first report of using ZFNs in generating GE pigs was to disrupt hemizygous *eGFP* gene. A pair of ZFNs could effectively inactivate the *eGFP* gene in porcine fibroblast cells through NHEJ. The efficiency of the ZFNs was around 5% [46]. The study showed that the DNA repair processes used for gene-editing systems are also present in pig somatic cells, thus, the use of ZFNs is possible in pigs. *PPARγ* was the first endogenous gene to be targeted using ZFN to develop GE pigs for a cardiovascular disease model [47]. In the study, efficiency of three designed ZFN pairs was tested by introducing them into parthenogenetically activated porcine oocytes by microinjection. One ZFN pair was selected from the screening and transfected into porcine cells to disrupt *PPARγ*. Then, heterozygous *PPARγ* knockout pigs were produced through SCNT. Generating knockout cells through the conventional gene targeting approach of using a targeting vector was extremely difficult due to the inefficiency in endogenous HR [32, 48]. However, these two studies demonstrated that ZFNs could effectively establish knockout cells without a targeting vector by relying on endogenous NHEJ system. Furthermore, ZFNs could also disrupt both alleles in pig cells. By transfecting ZFNs and phenotypically selecting α-Gal negative cells through fluorescence-activated cell sorting (FACS), *GGTA1* was effectively modified at biallelic fashion in porcine fibroblast cells and the cells were developmentally competent via SCNT [49]; the reported efficiency of targeting in this study was 1%. This was a significant achievement in GE pig production because previously only heterozygous modifications were possible through conventional gene targeting strategy.

Use of ZFNs could also lead to production of double knockout pigs without breeding. Two endogenous genes, *GGTA1* and *CMAH*, were inactivated by ZFNs in two-steps. First, both alleles of *CMAH* gene were disrupted in pig somatic cells. Then, ZFNs targeting *GGTA1* were transfected into the cells derived from *CMAH* knockout clones. Cells were counter-selected for the presence of α-Gal and *CMAH/GGTA1* double knockout cells lines were used to generate double knockout pigs through SCNT [50]. This was a significant improvement in xenotransplantation field as ability to disrupt multiples alleles and genes could reduce the number of breedings required to generate GE pigs suitable for xenotransplantation.

These reports utilized the endogenous NHEJ pathway after the DSBs, generated by ZFNs, to disrupt target genes. The first report of using HDR pathway to inactivate an endogenous gene was in 2013 [51]. We successfully disrupted *CMAH* in porcine fetal fibroblast cells by introducing plasmids coding for ZFNs, and a donor DNA carrying around 800 bp homology to the *CMAH* on each side and a selectable marker. The length of homology in the donor DNA was shorter compared to the conventional targeting vectors, indicating that the ZFN-induce DSBs could vigorously stimulate the HDR pathway. We also determined that donor DNA carrying longer homology arms resulted in higher frequency of HDR [51]. The cells were used to generate *CMAH* knockout pigs, showing in vivo competency of the approach.

The application of ZFNs dramatically reduced time required to generate GE pigs [52]. However, ZFNs also presented side effects such as off-site target cutting of the DNA and cytotoxicity, and it was challenging to assemble effective ZFNs pairs. The *Fok*I enzyme, the endonuclease of ZFNs, is supposed to be only activated when dimerized. However, studies demonstrated that *Fok*I could generate DSB at off-site targets as ZFNs combined with wild-type *Fok*I enzyme resulted in unintended DSBs [53–55].

#### TALEN

Transcription activator-like effector nucleases (TALENs) were developed from plant pathogenic bacteria in *Xanthomona* [56, 57]. Similar to the ZFN, TALENs need a string of TALEN motifs to bind to the specific locus of DNA on the genome, and *Fok*I enzyme acts as an endonuclease to introduce DSB. The binding domain of TALENs consists of a series of 33-35 amino acid repeats and this one TALEN motif can bind to a single base pair [58, 59]. TALENs provide more flexibility in target sequences because ZFNs are known to be more active towards GC-rich target regions, whereas TALENs can be assembled to target AT-rich regions and available TALEN kits made it easier to assemble effective TALEN sets [60].

TALENs have been successfully applied in GE pig production. In 2012, the first GE pigs generated using TALENs were reported [61]. The study showed that using a GoldyTALEN set, carrying truncated N- and C-terminal of TALEN, was more effective in inducing targeted mutations. The TALEN sets were also used to induce targeted mutations during embryogenesis via microinjection in pigs, although no GE pig was produced through this approach. As proof of concept, *LDLR* knockout pigs were produced through SCNT as a model of familial hypercholesterolemia disorder. The same group also demonstrated that HDR pathway could be successfully utilized during TALEN-mediated gene targeting [62]. TALEN plasmids or mRNA coding for TALENs were transfected into pig fibroblast cells with single-stranded donor DNA of varying lengths (40 - 100 nt). Interestingly, using TALEN mRNA resulted in a higher HDR efficiency than TALEN plasmid. Two different knockout pigs, *DAZL* and *APC*, were produced through SCNT to demonstrate in vivo competency of the cells. Intriguingly, we found out that the use of donor DNA could affect the frequency of NHEJ, indicating that there might be cross talk between molecules involved in NHEJ and HDR. The use of donor DNA with longer homology arms in TALEN-mediated gene targeting resulted in a higher percentage of knockout cells modified through NHEJ. [63]. The specific mechanism behind this observation is yet to be determined, but this suggests that the presence of donor DNA could stimulate DNA repair pathways.

Various types of GE pigs, models for xenotransplantation and muscle biology, were developed using TALENs as it could significantly increase the frequency of gene targeting [64–67]. The technology was also used to generate severe combined Immunodeficiency (SCID) pigs for stem cells transplantation study. We produced *RAG2* knockout pigs by TALENs and SCNT, and then introduced human induced pluripotent stem (hiPS) cells into the pigs. The pigs presented clear signs of a SCID phenotype and could support growth and differentiation of transplanted hiPS cells by forming teratomas [68]. This was the first report of teratoma formation from human stem cells using non-rodent models, demonstrating that pigs could be an excellent model for studying safety and efficacy in human regenerative medicine research.

#### CRISPR/Cas9

The CRISPR array was first reported in 1987. A series of arranged 29 nucleotides as direct repeats with 32 nucleotides as spacing were identified, although the exact function of this array was not determined at the time [69]. Later, this CRISPR array was characterized as an adaptive immune system of bacteria cells against exogenous DNA from virus or plasmid [70, 71]. The ability of the CRISPR/Cas9 system to induce DSBs on a specific sequence of DNA was adopted as an RNA-based gene-editing technology. Engineered single guide RNA (sgRNA) combined with tracr-RNA can bind to a target sequence, thus, locating Cas9 protein to the target site on the genome. Then, the Cas9 protein generates DSB to the target site if the protospacer adjacent motif sequence (PAM) is present at the locus [72]. Both ZFN and TALEN require assembling an array to make each set, which is complex and time-consuming [73, 74]. However, CRISPR/Cas9 system is easy to construct because only a 20-bp sgRNA needs to be inserted into a targeting vector [6]. Because of its user-friendly feature, the CRISPR/Cas9 system has become the leading gene-editing system. There is a concern of off-site cutting activity using CRISPR/Cas9 system because the system only requires 20 bp recognition [6, 72], and allows up to five base pair mismatches for the formation of DSB [75]. Preventative approaches such as using a modified Cas9, which induces a single-strand break instead of DSBs, has been suggested [6, 76].

The first application of CRISPR/Cas9 system to target genes in mammalian cells was in 2013 [6]. In pigs, the first use of CRISPR/Cas9 to produce GE pigs was by introducing the system into developing zygotes [77], which will be discussed in more detail in the following section. We first reported that CRISPR/Cas9 system could effectively introduce specific mutations in porcine fibroblast cells for GE pig production [8]. We also attempted to utilize HDR pathway in CRISPR/Cas9-mediated targeting system, although no colonies derived from HDR were identified. In 2015, it was demonstrated that two genes (*PINK1* and *PINK2*) could be simultaneously disrupted using CRISPR/Cas9 system in a single cell [78]; the frequency of multiplexing was 38.1% in the study. The multiplexing ability of CRISPR/Cas9 system seemed to be an ideal approach to inactivate multiple copies of porcine endogenous retrovirus (PERV) sequences in the pig genome; previous attempts to control PERV activity have not been successful [79–82]. Recently, two papers demonstrated that CRISPR/Cas9 system can effectively disrupt multiple copies of PERV in somatic cells and the cells could be used as a donor for SCNT to generate PERV-free pigs [83, 84], indicating that a potential major hurdle of using pigs for xenotransplantation has been lifted using CRISPR/Cas9 system.

The CRISPR/Cas9 system could utilize HDR pathway to place an exogenous DNA into a specific target site as a knock-in strategy [85, 86]. In 2015, a successful knock-in strategy was applied to integrate *GFP* gene into *pH 11* gene locus, a proposed safe harbor locus; ds-DNA containing 800 bp of homology arms on each side was used as a donor DNA. The efficiency of HDR was 54% with drug selection. Interestingly, only heterozygous gene knock-in events were observed. This is similar to our previous results using ZFNs [51], indicating higher activity of NHEJ can interfere with obtaining homozygous mutations through HDR.

### Direct injection of engineered endonucleases into zygotes to bypass the need for SCNT

Traditionally, GE pigs carrying site-specific modifications were produced through gene targeting in somatic cells, then SCNT was used to generate the animals. The process has been effective, however, a portion of animals born through this approach have typically had some developmental defects due to SCNT. Recent reports suggest that it is possible to introduce site-specific gene modification through introducing engineered endonucleases into developing embryos, thus, bypassing the need of SCNT.

#### Knock-out

TALENs were the first engineered endonucleases to be successfully used to introduce site-specific modifications without applying SCNT [87]. Pigs proposed to be resistant to African Swine Fever Virus were generated through this approach, demonstrating that SCNT is not necessary to introduce site-specific modifications in pigs. The direct injection approach was expanded with the development of CRISPR/Cas9 system, as it is simpler to assemble working sets of CRISPR/Cas9 system, compared to other engineered endonucleases. The first GE pigs generated using microinjection of *Cas9* mRNA and sgRNAs were *vWF* disrupted pigs [77]). The study reported that *Cas9* mRNA and sgRNA had low cytotoxicity during embryo development; embryo development was similar compared to water-injected embryo. The targeting efficiency through the microinjection was 68% among piglets born in the study. In the same year, we reported that microinjection of CRISPR/Cas9 system could result in 100% targeting efficiency [8]. We demonstrated that the approach could generate founders without carrying wild-type allele. The efficacy of CRISPR/Cas9 system during embryogenesis was examined using two genes, *CD163* and *CD1D*. Compared to the previous report, we were able to disrupt all wild-type alleles with a lower concentration of CRISPR/Cas9 RNA (10 ng/μL of sgRNA and *Cas9* mRNA); higher concentration of the RNA was toxic for embryo survival in this experiment. This could be beneficial as a previous report suggests that lower concentration of RNA help normal development and survival of CRISPR/Cas9 injected embryos [88].

Following studies reported that the microinjection of CRISPR/Cas9 system was effective in producing GE pigs. In 2015, *MITF* knockout pigs were produced by introducing CRISPR/Cas9 system into in vivo-derived embryos to serve as a melanoma model [89]. Because mature oocytes are transcriptionally inactive, the RNA-based CRISPR/Cas9 system is typically injected into developing embryos. However, it was demonstrated that a plasmid coding for CRISPR/Cas9 could also be effective in generating knockout pigs [90]. *GGTA1* knockout pigs for xenotransplantation were produced through this approach; three out of six piglets lacked functional *GGTA1* alleles. CRISPR/Cas9 system is also effective in introducing mutations on multiple genes. We demonstrated that the system could disrupt two genes simultaneously at near 100% efficiency in vitro [8]. The first report of pigs carrying multiple genes was reported in 2016 where *parkin/DJ-1/PINK1* were disrupted in an inbred line of pigs using in vivo derived zygotes [91]. Two piglets were born alive and both piglets carried modified target genes but one piglet carried one wild-type allele of *parkin*. Recently, we reported that the CRISPR/Cas9 system could effectively disrupt two target genes at 100% targeting efficiency; thus, founder animal could be used for viral challenge studies [7]. In this study, we used in vitro matured oocytes and in vitro fertilized embryos to generate *RAG2/IL2RG* double knockout pigs. To reduce the cytotoxicity associated with CRISPR/Cas9, we introduced a low concentration of sgRNA (2.5 ng/μL) and *Cas9* mRNA (5 ng/μL) after optimizing the system.

One of the main concerns related to direct injection of the CRISPR/Cas9 system is the resulting mosaic genotypes. This approach results in high incidents of mosaicism (20-70%) in founder rodents and has caused complications in analyzing phenotype of the founders [92–94]. However, only 10-20% of pigs generated in our previous studies presented a mosaic genotype [7, 8]. This difference between rodents vs. pigs is not characterized but could be due to disparity in embryo development [95, 96] or efficiency of sgRNA and *Cas9* mRNA used in each study.

As shown here, the direct injection of CRISPR/Cas9 system is effective in generating GE pigs. However, in most cases, in vivo derived oocytes or embryos have been used due to inefficiency in pig in vitro maturation (IVM) and fertilization (IVF). There are only a few papers demonstrating that the approach is possible using in vitro-derived oocytes [7, 8, 97]. In addition, indels introduced through NHEJ do not always result in disruption of target genes. If indels are in triplets, the function of target genes could be retained [7]. With the use of the HDR pathway or further optimization, the issues associated with microinjection approach could be minimized. A recent study demonstrates that high mutagenesis ability of the microinjection approach can also be applied to SCNT embryos [98]. This study showed that by introducing CRISPR/Cas9 system into cloned zygotes, high frequency of targeting was obtained; 100% biallelic modification in fetuses (6/6) was reported. Introducing CRISPR/Cas9 system into SCNT embryos can be powerful in causing mutations to a specific line of genetic background and reducing the effort required to identify cells carrying targeted modifications.

#### Knock-in

The CRISPR/Cas9 system can also stimulate HDR pathway to introduce site-specific modifications at the nucleotide level, when introduced into developing embryos. The HDR based knock-in strategy was first demonstrated by using parthenogenetic embryos in pigs [89]. Over 13% of the embryos were targeted through HDR by using single-stranded DNA (ssDNA) oligonucleotides with 26 bp homology on each side as a donor. The study also reported that the efficiency of knock-in was highly dependent on the concentration of donor DNA and sgRNA; no HDR event was observed under a lower concentration. In contrast, another study reported that higher concentration of ssDNA could decrease the frequency of HDR-derived modifications in vivo [99]. In the same year, the first successful application of HDR using double-stranded DNA (dsDNA) to generate GE pigs was announced [100]. This study used dsDNA carrying 1 kb of homologous sequence on each side as a donor DNA to integrate human albumin cDNA into the pig albumin locus. A total of 16 piglets were produced and human albumin gene was detected from all the piglets as a knock-in event.

As mentioned earlier, the frequency of NHEJ is typically higher in most cells compared to that of HDR. A recent report indicates that the use of NHEJ inhibitor can increase the frequency of embryos targeted through HDR [101]. By incubating CRISPR/Cas9 injected embryos with SCR7, a DNA ligase IV inhibitor, the efficiency of HDR was 100% in parthenogenetic embryos; but no incident of homozygous HDR-derived modifications was reported. The efficiency of knock-in was 40-60% without the inhibitor. This improvement in the frequency of HDR by the use of an NHEJ inhibitor is consistent with previous reports in rodents [102, 103].

**Fig. 1:**
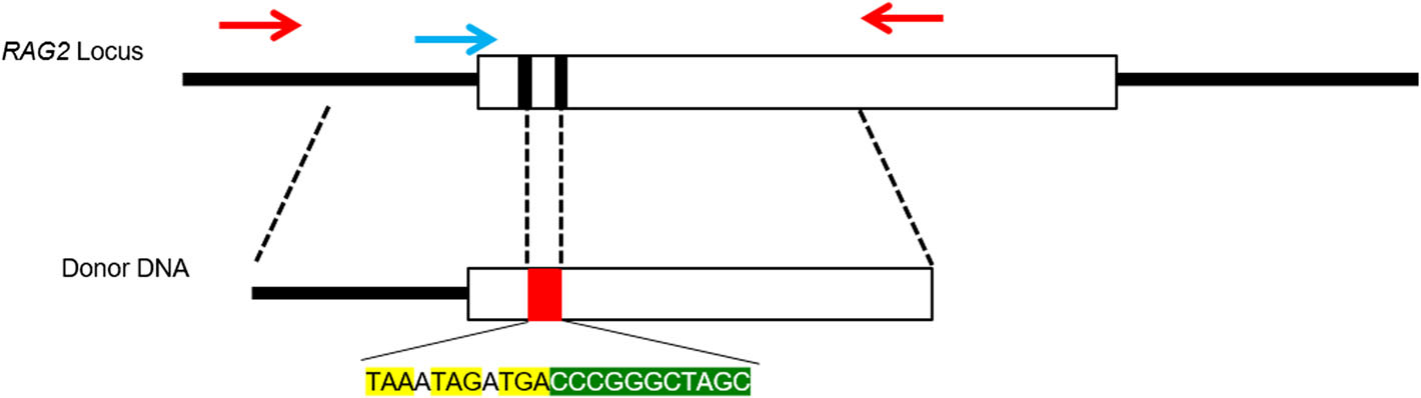
Strategy of inducing HDR during embryogenesis to disrupt *RAG2*. Two black bars indicate target sites by CRISPR/Cas9 system on *RAG2* exon. Red bar on the donor DNA shows the location of sequences introduced through HDR; yellow sequences are stop codons and green sequences are restriction enzyme sites (*Nhe*I and *Sma*I). Red arrows indicate the location of primers used to amplify the region for genotyping. Blue arrow was used as a primer for Sanger sequencing

**Fig. 2:**
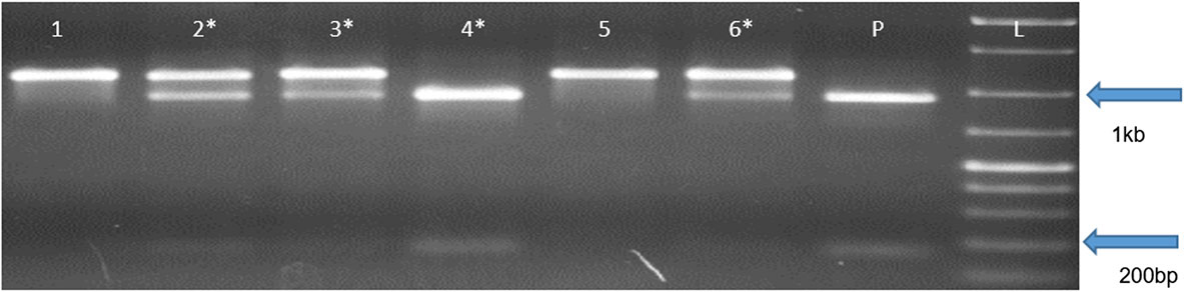
Genotyping results from single blastocysts injected with CRISPR/Cas9 system. All PCR products were digested with *Nhe*I. PCR product size from wild-type genomic DNA was 1.1 kb. If the embryo carried modified allele through HDR, we expected to see two fragments (950 bp and 160 bp) after digestion with *Nhe*I. Genomic DNA from an embryo carrying homozygous HDR mutation was served as a positive control (P). * indicates embryos carrying knock-in events. L is a molecular ladder

**Fig. 3:**
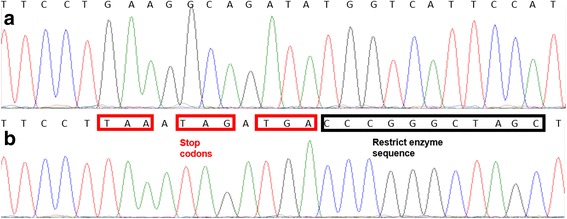
Chromatogram of genotyping results from (**a**) wild-type control and (**b**) embryo carrying knock-in sequence. Direct sequencing of PCR product indicates that this embryo (**b**) contains homozygous HDR alleles; introduced stop codons and restriction enzyme sequences are highlighted

These publications show that utilizing HDR pathway is possible during embryogenesis in pigs, although only a limited number of publications are available related to this topic. When we tested the efficiency of HDR using *RAG2* sgRNAs, previously used to produce *RAG2/IL2RG* double knockout pigs, we were able to utilize HDR to introduce specific mutations to the *RAG2* locus. A donor DNA containing 800 bp of homologous arms flanking designed stop codons and restrict enzyme sequences [Fig. 1] were introduced with CRISPR/Cas9 RNA into presumptive zygotes. Then, the injected blastocysts were lysed to extract DNA on d 7 post-IVF and PCR was used to identify knock-in events. Sanger sequencing and restriction enzyme digestion of the PCR products indicated that knock-in was successful [Figs. 2 and 3]; the overall efficiency of knock-in was 39.1% with 8.7% of the embryos carrying homozygous knock-in alleles (Table 1).

**Table 1:** A summary of HDR-derived gene-editing on *RAG2* locus. This is a summary of three independent replicates. A total of 154 embryos were injected and 28 embryos reached blastocysts on d 7. From 23 blastocysts genotyped, two embryos presented homozygous HDR-derived alleles (8.69%) and seven embryos carried heterozygous HDR-derived alleles (30.4%)

Type of gene-editing events	Frequency, %
Homozygous HDR	8.69
Heterozygous HDR	30.4
No HDR	60.8

All of the examples above exhibit the power of gene-editing systems in producing GE pigs. Proper application of gene-editing systems will effectively reduce the time required to generate GE pigs carrying targeted modifications, thus broadening the use of pig models in biomedicine and agriculture.

## Conclusion

Pig models are becoming a leading gene-edited biomedical model because they are physiologically, anatomically and genetically similar to humans. Rapid generation of GE pigs by utilizing gene-editing technology reduces the cost of housing for the pigs and number of breedings required to obtain enough animals. A recent study shows that gene-editing and gene-stacking technology can efficiently generate pigs carrying multiple knockout genes to serve as a model for xenotransplantation [104]. It would take decades to generate these type of pigs through conventional genetic engineering technology. We also demonstrated that founder GE pigs could be used for a viral challenge study; no herd of GE pigs was maintained to produce sufficient number of GE pigs [7]. The gene-editing technology has changed the way GE pigs are produced; however, there are still shortcomings or concerns related to this approach. Off-site editing can be a concern if design of sgRNA is not ideal. Modifications through NHEJ is hard to predict because the outcome of the modifications is random. Mosaic genotypes generated through direct injection of engineered endonuclease into zygotes could lead to founders with unexpected phenotypes. A number of strategies have been suggested to overcome these shortcomings. Use of Cas9 nickase, modified to introduce only single-stranded breaks, was proposed to minimize complications associated with DSBs [6, 105]. In addition, recent publications demonstrate that application of Cpf1, another CRISPR/Cas system adopted from another bacterial system, can provide higher diversity to target sequences to overcome limitations of designing effective sgRNAs for the CRISPR/Cas9 system [106]. These advancements in gene-editing technology will further expand the use of pig models in biomedicine and beyond.

## Abbreviations

CRISPR/Cas9: Components in the Clustered regularly interspaced short palindromic repeat /CRISPR-associated
DSB: Double-strand breaks
ES: Embryonic stem cells
GE: Genetically engineered
GFP: Green fluorescent protein
HDR: Homology direct repair
HR: Homologous recombination
Indel: Insertions or deletions
iPS: Induced pluripotent stem cells
IVF: in vitro fertilization
IVM: in vitro maturation
NHEJ: Non-homologous end joining
PAM: Protospacer adjacent motif sequence
SCID: Severe combined Immunodeficiency
SCNT: Somatic cell nuclear transfer
sgRNA: Single guide RNA
TALENs: Transcription activator-like effector nucleases
tracr-RNA: Trans-activating crRNA
ZFNs: Zinc-Finger Nucleases
